# Differentiation of Trafficking Pathways at Golgi Entry Core Compartments and Post-Golgi Subdomains

**DOI:** 10.3389/fpls.2020.609516

**Published:** 2020-12-08

**Authors:** Yoko Ito, Yohann Boutté

**Affiliations:** Laboratoire de Biogenèse Membranaire, Université de Bordeaux, Villenave d’Ornon, France

**Keywords:** membrane traffic, subdomains, secretion, endocytosis, lipids, trans-Golgi network, Golgi apparatus, protein sorting

## Abstract

Eukaryotic cells have developed specialized membrane structures called organelles, which compartmentalize cellular functions and chemical reactions. Recent improvements in microscopy and membrane compartment isolation techniques are now sophisticating our view. Emerging evidences support that there are distinct sub-populations or subdomains, which are spatially and/or temporally segregated within one type of organelle, contributing to specify differential sorting of various cargos to distinct destinations of the cell. In plant cells, the Golgi apparatus represents a main trafficking hub in which entry occurs through a Golgi Entry Core Compartment (GECCO), that remains to be further characterized, and sorting of cargos is mediated through multiple transport pathways with different sets of regulator proteins at the post-Golgi compartment *trans*-Golgi network (TGN). Both GECCO and TGN are differentiated sub-populations as compared to the rest of Golgi, and moreover, further subdomain formation within TGN is suggested to play a key role for cargo sorting. In this review, we will summarize recent findings obtained on organelle subdomains, and their relationship with cargo entry at and exit from the Golgi apparatus.

## Introduction

Membrane trafficking in eukaryotic cells substantially contributes to tissue and whole organism patterning by secretion to the extracellular space or controlling protein localization at the plasma membrane (PM) sometimes in a polar way at proper timings. The secretory trafficking partly relies on the Golgi apparatus, which consists of multiple flat cisternae piled up to form stacks in most eukaryotic cells including plants. Those cisternae are polarized across the stack between the *cis* side, which receives materials from the endoplasmic reticulum (ER), and the *trans* side, which sends them forward to their destinations. In addition to these flat cisternae, morphological studies in mammalian cells demonstrated that there are vesicular-tubular structures both on the *cis* and *trans* ends of the stack, which are suggested to be the specialized compartments for cargo sorting at the entry and exit sides of the Golgi (Griffiths and Simons, 1986; Mellman and Simons, 1992). The one at the *trans* side was named *trans*-Golgi network (TGN), and now its dynamics in plant cells is attracting interests from wide range of scientists in the trial to understand the complex sorting mechanisms taking place in this compartment. In addition, it clearly appears now that TGN is further divided into subdomains or sub-populations whose composition, dynamics and function remains to be fully deciphered. The one at the *cis* side, which was named ER-Golgi intermediate compartment (ERGIC) in mammalian cells, was not recognized in plants before, but recent studies are revealing the existence of specialized compartment at the ER-Golgi interface in plant cells as well.

## The Golgi Entry Face: *cis*-Compartments As The ER-Golgi Ferryman

**Figure 1:**
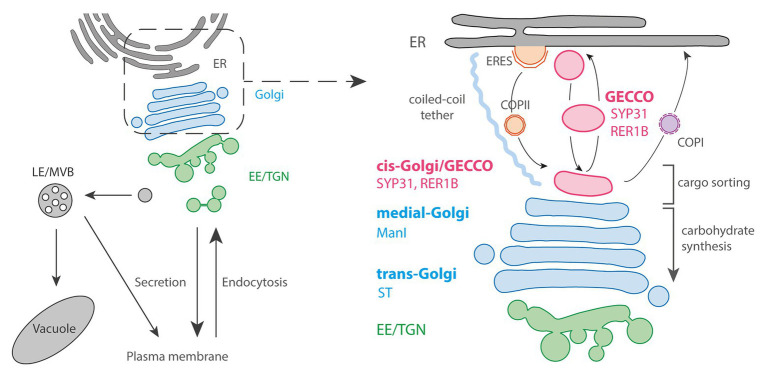
The Golgi entry face. **Left panel:** general schematic representation of the endomembrane trafficking starting with ER: Endoplasmic Reticulum followed by Golgi and post-Golgi trafficking with the Early Endosome (EE)/*trans*-Golgi Network (TGN), the Late Endosome (LE)/Multi-Vesicular Bodies (MVBs), vacuole and plasma membrane (PM). **Right panel:** the pre-Golgi trafficking involves COPII-anterograde transport through Endoplasmic Reticulum Export Sites (ERES) as well as COPI-retrograde transport. The Golgi Entry Core Compartment (GECCO) is a compartment independent from the COPII machinery, and could function as an intermediate compartment between ER and Golgi. GECCO shares its localizing proteins (SYP31 and RER1B) with the *cis*-most Golgi cisternae. GECCO and the first *cis*-Golgi cisternae might be specialized in cargo sorting, while carbohydrate synthesis occurs only from *medial* and *trans* cisternae due to the specific localization of glycosylation enzymes (e.g., ManI or sialyltransferase; ST) at these later cisternae. The Golgi stacks are physically tethered to the ER by long coiled-coil tethering elements.

The ER produces COPII vesicles at specialized domains called ER exit/export sites (ERES) for anterograde trafficking to the Golgi and the Golgi sends ER components back by COPI vesicles to the ER (Figure 1). In spite of this continuous communication between the ERES and Golgi, about a half of total ERES are located far from the Golgi in vertebrate cells (Stephens, 2003). Instead of direct ER-Golgi interaction, some vesicular-tubular structures are obviously separated from the other Golgi cisternae in vertebrate cells, and now recognized as the pre-Golgi compartment that receives cargoes from the ER before the *cis*-Golgi. It is commonly termed as Vesicular Tubular Cluster (VTC), ER-Golgi Intermediate Compartments (ERGIC), or just intermediate compartment (IC). Since it contains both anterograde and retrograde cargos (Palokangas et al., 1998; Simpson et al., 2006), depends on both COPI and COPII machineries (Scales et al., 1997), and furthermore, provided that some protein sorting receptors cycle between the ER and ERGIC (Dancourt and Barlowe, 2010), ERGIC is thought as the place for sorting between the ER and Golgi. Recently, a new model suggests that ERGIC can be divided into stationary globular domain, which is associated with ERES, and dynamic tubular domain which protrudes from the globular domain. This is based on the observations that a GTPase RAB1 preferentially localizes to the tubular domain than the globular domain, while some other proteins including the ERGIC marker p58/ERGIC-53 show the opposite distribution, which indicates the differentiation of these domains (Sannerud et al., 2006; Saraste and Marie, 2018).

In plant cells, the Golgi stacks are dispersed throughout the cytoplasm with continuous association with ERES, although it has been observed that there are some free and small ERES without being associated with Golgi (Ito et al., 2014; Brandizzi, 2018; Takagi et al., 2020). In the associated pairs, ERES and Golgi are physically tethered by a long coiled-coil protein and keep the close distance during rapid movement along actin filaments (Osterrieder et al., 2017). Most of the COPII budding from the ER occurs within only 300 nm from the *cis*-Golgi, while the size of a single COPII bud in *Arabidopsis* is approximately 60 nm in diameter (Kang and Staehelin, 2008; Staehelin and Kang, 2008). Due to these constraints, an “ERGIC-like structure” has never been morphologically determined within this limited space. However, electron tomography has demonstrated that the size and shape of the very first *cis* cisternae are tremendously variable from a small “blob,” with the size corresponding to only several vesicles, to branched tubules or disc-shaped cisternae, suggesting that they are in the course of cisternal assembly (Staehelin and Kang, 2008). *α*-1,2-Mannosidase I (ManI), the first enzyme that works in the *N*-glycosylation reaction of proteins at Golgi, was revealed to mainly localize at the third and fourth cisternae, not to the first and second ones by immuno-electron tomography (Donohoe et al., 2013). Since the glycosylation enzymes are distributed within the Golgi largely in the order that they act, this data indicates that the cisternae at the most *cis* side in plant cells are biochemically inactive, similarly to mammalian ERGIC.

Live-cell observations have also demonstrated that the *cis*-most cisternae have a distinct nature from the others cisternae in plant cells. Brefeldin A, a drug that inhibits the activation of ARF1 GTPase, causes Golgi disassembly and relocalization of Golgi enzymes to the ER in many organisms and cell types including tobacco cultured cells (Lippincott-Schwartz et al., 1989; Ritzenthaler et al., 2002). In tobacco BY-2 cultured cells, *cis*-Golgi proteins SYP31 and RER1B were found to localize at unknown punctate structures close to ERES upon brefeldin A (BFA) treatment, while other Golgi markers including ManI were distributed in the ER (Ito et al., 2012, 2018). In addition, because the punctate structures labeled by SYP31 received other Golgi components from the ER and the Golgi stacks were regenerated from them after removal of BFA, these structures can be thought as the entry site of the Golgi and the scaffold for stack assembly. Thus, the structure was given the name “Golgi entry core compartment” (GECCO; Figure 1; Ito et al., 2012, 2018). In mammalian cells, an ERGIC marker protein ERGIC-53 shows a similar behavior upon BFA treatment by localizing to punctate structures called Golgi remnants (Lippincott-Schwartz et al., 1990). Therefore, GECCO that appears by BFA treatment would be corresponding to the Golgi remnants, and the *cis*-most cisternae in plant cells would be the counterpart of ERGIC. However, contrastingly to the mammalian ERGIC-53, which is trapped in the ER by the expression of a dominant mutant of SAR1 GTPase, the localization of SYP31 to GECCO in tobacco BY-2 cells is not affected by SAR1 dominant mutant, indicating that ER-to-GECCO transport independent from COPII machinery exists in plant cells (Shima et al., 1998; Hauri et al., 2000; Ito et al., 2018). In *Saccharomyces cerevisiae*, although it is impossible to define pre-Golgi compartment by spatial relationship among cisternae, 3D live-cell imaging has revealed that only the *cis* cisternae approach to ERES and contact transiently to receive COPII-mediated cargos (“hug-and-kiss” action; Kurokawa et al., 2014). Considering their function as the Golgi entry compartment, the cisternae that show this hug-and-kiss action would correspond to mammalian ERGIC and plant *cis*-Golgi/GECCO. If the COPII-mediated transport occurs by hug-and-kiss also during the formation of new *cis* cisternae, some preexisting compartment should be in front of the ER to capture the first COPII carriers. The finding of GECCO as a COPII-independent structure in plants might contribute to understand the process of cisterna initiation.

## The Golgi Exit Face: The Multifaceted TGN Is Differentiated in Functional Subdomains

The TGN is generally defined as a vesicular-tubular structure at the *trans* side of Golgi. In plants, 3D tomographic studies showed that the *trans*-most cisternae seem to peel off from the stack and mature by changing their morphology from early TGN with central flat domain into late TGN with many vesicles connected by tubules (Staehelin and Kang, 2008; Kang et al., 2011). It is also reported in *Arabidopsis* that impaired TGN biogenesis in the *lot* (loss of TGN, a Golgi-localized putative activator for the small GTPase Rab6) mutant is accompanied by the overstacking of the Golgi, supporting that the TGN is generated by the maturation of the Golgi cisternae (Jia et al., 2018). However, in tobacco cells, pharmacological induction of the disassembly and reassembly of the TGN suggested that TGN biogenesis would not fully depend on the cisternal maturation from the *trans*-Golgi (Ito et al., 2017).

Once formed, TGN are able to further differentiate into other compartments of different composition. A striking example is the differentiation of TGN into pre-vacuolar compartments (PVCs)/multi vesicular bodies (MVBs; Scheuring et al., 2011). Other examples are the compartments labeled by either RAB-A5c or RAB-A4b. In both cases, these compartments are thought to be derived from TGN but at some point they become so differentiated that they do not co-localize any longer with any TGN or other known endomembrane compartments markers (Preuss et al., 2004; Kirchhelle et al., 2016).

The multiple identity of TGN is also seen at the level of trafficking routes crossing the TGN. The TGN is known to not only receive secretory cargos from the Golgi but also cargos from the endocytic pathway. In mammalian cells, the early endosomes (EE) first receive the proteins endocytosed from the PM, and some of those proteins are recycled back to the PM directly from the EE or *via* tubular compartments called the recycling endosomes (RE), or transported to the late endosomes/multi vesicular bodies (LE/MVB) generated by the maturation of the EE (Naslavsky and Caplan, 2018). The TGN exchanges materials with them, and some endocytic cargos are known to reach the TGN *via* those endosomes (Bonifacino and Rojas, 2006; Makaraci and Kim, 2018). The TGN in plant cells also receives endocytic cargos. However, by contrast to mammalian cells, since the endocytic tracer FM4-64 reaches the TGN within a few minutes before accumulating at the LE/MVB, it is thought that the TGN itself is equivalent to the EE in plant cells (Dettmer et al., 2006; Viotti et al., 2010). A recent study revealed that the TGN receives some proteins and FM4-64 endocytosed from the PM before the LE/prevacuolar compartment in budding yeast as well, suggesting the function of the TGN as the EE in the ancestral membrane trafficking system (Day et al., 2018). The mechanisms that achieve this complex cargo receiving and sorting at the TGN are largely unknown. However, recent studies are revealing that the division of roles between sub-populations of TGN contributes to those sorting processes.

**Figure 2:**
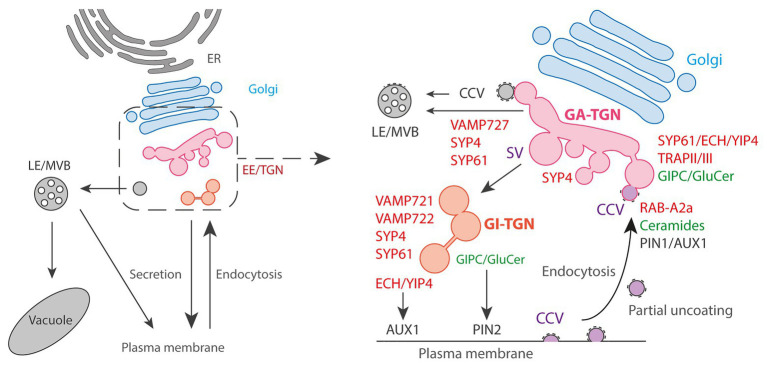
The Golgi exit face. **Left panel:** general schematic representation of the endomembrane trafficking starting with ER: Endoplasmic Reticulum followed by Golgi and post-Golgi trafficking with the EE/TGN, the LE/MVBs, vacuole and PM. **Right panel:** the post-Golgi trafficking is complex and involves multiple pathways interconnected at TGN. Golgi-Associated (GA) TGN can mature and detach from Golgi to become a Golgi-independent (GI) TGN. Trafficking from TGN to LE/MVBs is thought to partly rely on Clathrin-Coated Vesicles (CCVs) and the SNARE VAMP727. Presence of CCVs at TGN is also attributed to the endocytic pathway from the PM due to the partial uncoating of endocytic clathrin-coated vesicles that are collected by the EE/TGN. At clathrin/TGN subdomain, the small GTPase RAB-A2a is involved in PIN1 and AUX1 endocytic sorting through the action of the sphingolipid ceramides. At Secretory Vesicles (SVs)/TGN, the ECH/YIP4 complex is involved in AUX1 secretory sorting to PM while the sphingolipids GIPC/GluCer are involved in PIN2 secretory sorting to PM.

At the dynamic level, live-cell observations have demonstrated that TGN can either be associated with the *trans* side of the Golgi apparatus (Golgi-associated TGN/GA-TGN), or can be disassociated and moves independently from the Golgi (Golgi-independent TGN/GI-TGN or free TGN; Figure 2; Viotti et al., 2010; Kang et al., 2011; Uemura et al., 2014). GA-TGN and GI-TGN can undergo homotypic or heterotypic fusion and fission, and GI-TGN sometimes associates with the Golgi apparatus transiently, indicating that they might exchange materials with each other (Viotti et al., 2010).

These two types of TGN sub-populations are labeled by the syntaxin SYP43 (SYntaxin of Plants43), a component of a SNARE (Soluble *N*-ethylmaleimide sensitive factor Attachment protein REceptor) complex localized at TGN. Most of the SYP43 labels GA-TGN co-localize with the SNAREs VAMP721, VAMP722, and VAMP727. Although these three VAMP7 proteins are close homologs, it is known that VAMP721 and VAMP722 are involved in the trafficking to the PM and cell plate while VAMP727 mainly functions in the trafficking to vacuoles (Ebine et al., 2008; Kwon et al., 2008; Zhang et al., 2011; El Kasmi et al., 2013). In contrast to the GA-TGN, GI-TGN shows less co-localization with VAMP727, whereas VAMP721 and VAMP722 still co-localize well with GI-TGN. In addition, the GI-TGN co-localize with FM4-64 much less compared to the GA-TGN, indicating that the GI-TGN is more specialized to the trafficking to the PM as compared to the vacuolar trafficking or endocytic recycling (Uemura et al., 2019). This is consistent with a previous report that CONTINUOUS VASCULAR RING (COV1), a TGN-localized protein which is required for vacuolar protein sorting, is involved in the association of the GA-TGN to the *trans*-most cisternae of the Golgi (Shirakawa et al., 2014). In contrast, ECHIDNA (ECH) and its interactors YPT/RAB GTPase Interacting Protein 4a (YIP4a) and YIP4b are also known to localize to the TGN and contribute to the proper association between the Golgi and GA-TGN, but they function in the secretory transport of specific proteins and polysaccharides to the PM, not in the vacuolar or endocytic trafficking (Gendre et al., 2011, 2013). This might indicate that the trafficking routes of SYP4-VAMP721 and ECH-YIP4 are differentiated within the TGN. Although it is not yet clear, the GA‐ and GI-TGN are also presumed to be involved in plant growth and development differently from the fact that the GI-TGN is found more frequently in root differentiation zone compared to meristematic zone (Uemura et al., 2014).

Besides, it also becomes increasingly clear that TGN vesicles are morphologically and functionally diverse. Originally, subdomains at the TGN were first suggested by immunogold electron microscopy which found that the TGN SNAREs SYP41 and SYP42 localized to distinct parts of the same TGN (Bassham et al., 2000; Sanderfoot et al., 2001). Later on, it was observed that two TGN-localized proteins, the vacuolar V-ATPase VHA-a1 and the Rab GTPase RAB-A2a only weakly overlap by confocal microscopy (Chow et al., 2008). From electron tomography analyses, it is clear that at least two populations co-exist at TGN according to that they are either coated with clathrin for CCVs (clathrin coated vesicles) or uncoated for secretory vesicles (SVs; Figure 2; Kang et al., 2011; Boutté et al., 2013; Wattelet-Boyer et al., 2016). Electron microscopy analyzes revealed that VHA-a1 and the syntaxin SYP61 localize at SVs (Kang et al., 2011). Confocal microscopy additionally revealed strong co-localization of the protein ECHIDNA with the SVs markers VHA-a1 or SYP61 while ECHIDNA or VHA-a1 displayed only weak co-localization with clathrin (Gendre et al., 2011; Boutté et al., 2013). Oppositely, RAB-A2a displays strong co-localization with clathrin compared to SYP61 (Doyle et al., 2015; Wattelet-Boyer et al., 2016). These results suggest that VHA-a1/SYP61/ECHIDNA are present in one subdomain of TGN while RAB-A2a/clathrin would constitute another subdomain of TGN. The presence of CCVs at TGN was first attributed to involvement of clathrin-mediated trafficking from TGN to vacuoles (Sanderfoot et al., 1998; Sauer et al., 2013; Reynolds et al., 2018). However, recently, an elegant study revealed that CCVs at TGN also originate from the endocytic trafficking (Figure 2; Narasimhan et al., 2020). Interestingly, while in mammalian cells the CCVs formed at PM shortly release their clathrin coat, CCVs of plant cells get only partially uncoated while being collected by EE/TGN at close proximity of PM (Narasimhan et al., 2020). Consistent with the endocytic nature of CCVs at TGN, RAB-A2a‐ colocalizes with the endocytic tracer FM4-64 within a couple of minutes after external application (Chow et al., 2008). Partially uncoated CCVs could be a central place of recycling at TGN and a way for TGN to segregate the endocytic recycling material from the *de novo* secretory material. However, further investigations are required to decipher the complete role and dynamics of CCVs at TGN (Reynolds et al., 2018). Similarly, the function of SVs at TGN attracts increasing attention. As CCVs, SVs are thought to play a role during early stages of endocytosis based on the accumulation of FM4-64 a few minutes after application (Dettmer et al., 2006; Viotti et al., 2010). This is conceivable due to the partial fusion of CCVs with EE/SVs and the non-specific lipophilic nature of FM4-64. At the functional level, ECHIDNA and VHA-a1 are involved in cell elongation while RAB-A2a or DYNAMIN-RELATED PROTEINS (DRPs) are involved in cell division. Consistently, RAB-A2a or DRPs localize to the cell plate while ECHIDNA or VHA-a1 do not (Dettmer et al., 2006; Chow et al., 2008; Konopka et al., 2008; Boutté et al., 2010; Gendre et al., 2011). However, it would be hasty and oversimple to state that the division of labor between cell elongation and cell division is supported by either SVs or CCVs. The localization of CCV-proteins at the cell plate probably reflects the contribution of the endocytosis to the building of new membranes, which is possible considering the partial uncoating of CCVs, and/or it reflects the vesicular recycling from the middle to the edges of the cell plate as the division plane is extending (Chow et al., 2008; Richter et al., 2014). Moreover, RAB-A2a is not only involved in cell division but also in cell elongation (Chow et al., 2008; Li et al., 2017). Similarly, SVs do not contribute only to cell elongation but are also involved in the building of the cell plate by delivering *de novo* synthesized materials (Reichardt et al., 2007; Richter et al., 2014). For example, the syntaxin SYP61 and the TRS120 subunit of the Transport Protein Particle II (TRAPP II) tethering complex both localize to SVs and at the cell plate (Kang et al., 2011; Ravikumar et al., 2018). Interestingly, TRAPPII and ECHIDNA co-localize very well at SVs but are involved in two distinct cellular processes as loss-of function of *ECHIDNA* does not lead to obvious cell division defect contrarily to loss-of function of *TRS120*/*TRAPPII* (Ravikumar et al., 2018). Moreover, an element of a TRAPPIII complex has been shown to play a role at TGN in a yet different trafficking route involved in endocytic trafficking (Rosquete et al., 2019). Thus, trafficking pathways at SVs are diverse and rely on distinct molecular machineries.

Another striking evidence for such a sub-compartmentalization is coming from the lipid composition of these compartments. Immuno-isolation of SYP61-positive TGN and RAB-A2a-positive TGN using the corresponding proteins as baits revealed a specific enrichment of *α*-hydroxylated VLCFAs (hVLCFAs) at SYP61-TGN but not RAB-A2a-TGN, as compared to Golgi (Wattelet-Boyer et al., 2016). hVLCFAs are a specific signature of sphingolipids (SLs) and enrichment of sterols was also detected at SVs, which suggest that SLs and sterols could form small lipid platforms within SVs to sort specific cargos (Boutté et al., 2010; Wattelet-Boyer et al., 2016). Interestingly, while hVLCFAs of the final form of SLs, namely glucosylceramide (GluCer) and glycosylinositolphosphorylceramides (GIPCs), have been shown to play a role in secretory sorting of the efflux auxin carrier PIN2 at SVs, they do not act in secretory sorting of the auxin efflux carrier PIN1 or the auxin influx carrier AUX1 (Figure 2; Wattelet-Boyer et al., 2016). Contrastingly, ECHIDNA which is localized at SVs is involved in secretory sorting of AUX1 but not PIN1 or PIN2 (Figure 2; Gendre et al., 2011; Boutté et al., 2013; Jonsson et al., 2017). Intriguingly, intermediate forms of SLs, namely the ceramides, play a role in endocytic trafficking of PIN1 and AUX1 at RAB-A2/clathrin compartments, but not PIN2 (Figure 2; Markham et al., 2011). Hence, we should not think of TGN as a single homogeneous population. Even within SVs, protein cargos are segregated according to specific protein‐ and lipid-mediated sorting mechanisms.

## Conclusion and Outlook

It is clear that Golgi trafficking can no longer be seen as a general bulk flow. Both pre‐ and post-Golgi are divided into subdomains defining distinct protein sorting mechanisms and trafficking pathways. This subdivision represents most likely a phenomenon commonly found in endomembrane trafficking. Indeed, the rims or even a part within the rim region of the Golgi cisternae were also suggested to function as specialized subdomains based on the concentrated localization of specific proteins, which might indicate the existence of a subdomain sorting system at the Golgi cisternae as well (Pimpl et al., 2000; Naramoto et al., 2014; Meents et al., 2019). In the future, the use of super-resolution techniques such as stimulated emission depletion (STED), structured illumination microcopy (SIM), or super-resolution confocal live imaging microscopy (SCLIM) will be essential to distinguish subdomains with enough resolution. Moreover, the complete biochemical characterization of pre‐ and post-Golgi subdomains, both at the proteins and lipids level, would be a great advance to decipher the mechanisms through which lipids and proteins synergistically act during maturation of subdomains and differentiation of trafficking pathways. Protein and lipid characterization of TGN subdomains has already been supported by several studies but requires further investigations. In contrast, GECCO vesicles have not been isolated yet, while this would help us to define the nature and dynamics of GECCO and would be an important key in understanding ER to Golgi sorting mechanisms. Moreover, in spite of the accumulating evidences as described in this mini-review, the cargo transport *via* the membrane subdomains has never been directly observed in plant cells due to the lack of microscopic resolution and techniques to visualize cargo proteins. The improvement in those techniques would bring us a breakthrough in the near future. Finally, the complexity of membrane compartmentalization has to be accounted for its function in cellular organization sustaining developmental processes. Although, we did not address it in this mini-review due to lack of space, this question is indeed central to understand how protein sorting and membrane sub-compartmentalization is acting across cellular and developmental scales.

## Author Contributions

YI and YB wrote and edited the manuscript and prepared figures. All authors contributed to the article and approved the submitted version.

### Conflict of Interest

The authors declare that the research was conducted in the absence of any commercial or financial relationships that could be construed as a potential conflict of interest.
